# Supplementary material for: New Evidence for the Bronze Age Zooarchaeology in the Inland Area of the Iberian Peninsula through the Analysis of Pista de Motos (Villaverde Bajo, Madrid)
Source: Animals (Basel). 2024 Jan 26;14(3):413. doi: 10.3390/ani14030413 (PMC10854931; doi:10.3390/ani14030413)
Supplement: Supplementary file 1 [file animals-14-00413-s001.zip › animals-2802754-supplementary.pdf]

1. **Supplementary Table S1. Relative chronological correspondence assigned to the Pista de Motos site.**

SU	Chronology	SU	Chronology	SU	Chronology
2301	Neolithic	111	Middle Bronze Age	811	Iron Age II
2491	Neolithic	121	Middle Bronze Age	2071	Iron Age II
**SU**	**Chronology**	191	Middle Bronze Age	2121	Iron Age II
431	Bell-Beaker Bronze Age	201	Middle Bronze Age	2171	Iron Age II
481	Bell-Beaker Bronze Age	241	Middle Bronze Age	2251	Iron Age II
571	Bell-Beaker Bronze Age	271	Middle Bronze Age	2451	Iron Age II
573	Bell-Beaker Bronze Age	311	Middle Bronze Age	2461	Iron Age II
681	Bell-Beaker Bronze Age	321	Middle Bronze Age	2501	Iron Age II
691	Bell-Beaker Bronze Age	356	Middle Bronze Age	2541	Iron Age II
721	Bell-Beaker Bronze Age	361	Middle Bronze Age	2941	Iron Age II
1251	Bell-Beaker Bronze Age	391	Middle Bronze Age	**SU**	**Chronology**
1771	Bell-Beaker Bronze Age	451	Middle Bronze Age	2063	Visigoths
1831	Bell-Beaker Bronze Age	461	Middle Bronze Age	2065	Visigoths
2201	Bell-Beaker Bronze Age	471	Middle Bronze Age	2227	Visigoths
2203	Bell-Beaker Bronze Age	501	Middle Bronze Age	2291	Visigoths
2236	Bell-Beaker Bronze Age	591	Middle Bronze Age	2396	Visigoths
2351	Bell-Beaker Bronze Age	601	Middle Bronze Age	2481	Visigoths
2417	Bell-Beaker Bronze Age	611	Middle Bronze Age	2601	Visigoths
2441	Bell-Beaker Bronze Age	621	Middle Bronze Age	**SU**	**Chronology**
2571	Bell-Beaker Bronze Age	631	Middle Bronze Age	2557	Islamic Age
2823	Bell-Beaker Bronze Age	636	Middle Bronze Age	2565	Islamic Age
**SU**	**Chronology**	661	Middle Bronze Age	2567	Islamic Age
101	Prehistorian Indet	663	Middle Bronze Age		
131	Prehistorian Indet	671	Middle Bronze Age		
141	Prehistorian Indet	711	Middle Bronze Age		
151	Prehistorian Indet	751	Middle Bronze Age		
161	Prehistorian Indet	761	Middle Bronze Age		
171	Prehistorian Indet	781	Middle Bronze Age		

181	Prehistorian Indet	791	Middle Bronze Age		
196	Prehistorian Indet	801	Middle Bronze Age		
211	Prehistorian Indet	821	Middle Bronze Age		
221	Prehistorian Indet	826	Middle Bronze Age		
231	Prehistorian Indet	831	Middle Bronze Age		
251	Prehistorian Indet	841	Middle Bronze Age		
256	Prehistorian Indet	851	Middle Bronze Age		
261	Prehistorian Indet	854	Middle Bronze Age		
281	Prehistorian Indet	856	Middle Bronze Age		
291	Prehistorian Indet	857	Middle Bronze Age		
331	Prehistorian Indet	870	Middle Bronze Age		
341	Prehistorian Indet	872	Middle Bronze Age		
351	Prehistorian Indet	873	Middle Bronze Age		
371	Prehistorian Indet	876	Middle Bronze Age		
381	Prehistorian Indet	878	Middle Bronze Age		
411	Prehistorian Indet	881	Middle Bronze Age		
421	Prehistorian Indet	886	Middle Bronze Age		
441	Prehistorian Indet	901-902	Middle Bronze Age		
476	Prehistorian Indet	911	Middle Bronze Age		
491	Prehistorian Indet	921	Middle Bronze Age		
511	Prehistorian Indet	931	Middle Bronze Age		
521	Prehistorian Indet	941	Middle Bronze Age		
531	Middle Bronze Age ?	951	Middle Bronze Age		
541	Prehistorian Indet	961	Middle Bronze Age		
551	Prehistorian Indet	963	Middle Bronze Age		
676	Prehistorian Indet	971	Middle Bronze Age		
701	Prehistorian Indet	981	Middle Bronze Age		
702	Prehistorian Indet	991	Middle Bronze Age		
713	Prehistorian Indet	1001	Middle Bronze Age		
723	Prehistorian Indet	1011	Middle Bronze Age		
731	Prehistorian Indet	1016	Middle Bronze Age		

741	Prehistorian Indet	1021	Middle Bronze Age		
771	Prehistorian Indet	1031	Middle Bronze Age		
859	Prehistorian Indet	1041	Middle Bronze Age		
861	Prehistorian Indet	1061	Middle Bronze Age		
1018	Prehistorian Indet	1081	Middle Bronze Age		
1071	Prehistorian Indet	1091	Middle Bronze Age		
1121	Prehistorian Indet	1101	Middle Bronze Age		
1131	Prehistorian Indet	1111	Middle Bronze Age		
1191	Prehistorian Indet	1161	Middle Bronze Age		
1211	Prehistorian Indet	1666	Middle Bronze Age		
1256	Prehistorian Indet	1171	Middle Bronze Age		
1261	Prehistorian Indet	1181	Middle Bronze Age		
1271	Prehistorian Indet	1201	Middle Bronze Age		
1281	Prehistorian Indet	1202	Middle Bronze Age		
1301	Prehistorian Indet	1204	Middle Bronze Age		
1306	Prehistorian Indet	1221	Middle Bronze Age		
1323	Prehistorian Indet	1222	Middle Bronze Age		
1401	Prehistorian Indet	1231	Middle Bronze Age		
1402	Prehistorian Indet	1241	Middle Bronze Age		
1421	Prehistorian Indet	1291	Middle Bronze Age		
1491	Prehistorian Indet	1311	Middle Bronze Age		
1511	Prehistorian Indet	1321	Middle Bronze Age		
1531	Prehistorian Indet	1331	Middle Bronze Age		
1571	Prehistorian Indet	1336	Middle Bronze Age		
1581	Prehistorian Indet	1341	Middle Bronze Age		
1611	Prehistorian Indet	1351	Middle Bronze Age		
1631	Prehistorian Indet	1361	Middle Bronze Age		
1671	Prehistorian Indet	1371	Middle Bronze Age		
1681	Prehistorian Indet	1381	Middle Bronze Age		
1736	Prehistorian Indet	1391	Middle Bronze Age		
1741	Prehistorian Indet	1403	Middle Bronze Age		

1751	Prehistorian Indet	1411	Middle Bronze Age		
1752	Prehistorian Indet	1423	Middle Bronze Age		
1761	Prehistorian Indet	1431	Middle Bronze Age		
1781	Prehistorian Indet	1436	Middle Bronze Age		
1796	Prehistorian Indet	1441	Middle Bronze Age		
1811	Prehistorian Indet	1451	Middle Bronze Age		
1881	Prehistorian Indet	1461	Middle Bronze Age		
1893	Prehistorian Indet	1470	Middle Bronze Age		
1895	Prehistorian Indet	1470-76	Middle Bronze Age		
1897	Prehistorian Indet	1476	Middle Bronze Age		
1901	Prehistorian Indet	1478	Middle Bronze Age		
1903	Prehistorian Indet	1481	Middle Bronze Age		
1941	Prehistorian Indet	1501	Middle Bronze Age		
1954	Prehistorian Indet	1521	Middle Bronze Age		
1971	Prehistorian Indet	1541	Middle Bronze Age		
2031	Prehistorian Indet	1551	Middle Bronze Age		
2111	Prehistorian Indet	1561	Middle Bronze Age		
2141	Prehistorian Indet	1591	Middle Bronze Age		
2151	Prehistorian Indet	1601	Middle Bronze Age		
2156	Prehistorian Indet	1621	Middle Bronze Age		
2211	Prehistorian Indet	1641	Middle Bronze Age		
2221	Prehistorian Indet	1651	Middle Bronze Age		
2231	Prehistorian Indet	1661	Middle Bronze Age		
2281	Prehistorian Indet	1665	Middle Bronze Age		
2311	Prehistorian Indet	1691	Middle Bronze Age		
2321	Prehistorian Indet	1701	Middle Bronze Age		
2326	Prehistorian Indet	1711	Middle Bronze Age		
2401	Prehistorian Indet	1731	Middle Bronze Age		
2414	Prehistorian Indet	1791	Middle Bronze Age		
2431	Prehistorian Indet	1821	Middle Bronze Age		
2511	Prehistorian Indet	1836	Middle Bronze Age		

2555	Prehistorian Indet	1841	Middle Bronze Age		
2561	Prehistorian Indet	1851	Middle Bronze Age		
2611	Prehistorian Indet	1861	Middle Bronze Age		
2621	Prehistorian Indet	1871	Middle Bronze Age		
2651	Prehistorian Indet	1891	Middle Bronze Age		
2652	Prehistorian Indet	1921	Middle Bronze Age		
2653	Prehistorian Indet	1923	Middle Bronze Age		
2666	Prehistorian Indet	1931	Middle Bronze Age		
2671	Prehistorian Indet	1951	Middle Bronze Age		
2701	Prehistorian Indet	1952	Middle Bronze Age		
2721	Prehistorian Indet	1961	Middle Bronze Age		
2742	Prehistorian Indet	1976	Middle Bronze Age		
2743	Prehistorian Indet	1981	Middle Bronze Age		
2745	Prehistorian Indet	1991	Middle Bronze Age		
2751	Prehistorian Indet	2001	Middle Bronze Age		
2776	Prehistorian Indet	2011	Middle Bronze Age		
2781	Prehistorian Indet	2051	Middle Bronze Age		
2782	Prehistorian Indet	2081	Middle Bronze Age		
2791	Prehistorian Indet	2091	Middle Bronze Age		
2796	Prehistorian Indet	2101	Middle Bronze Age		
2799	Prehistorian Indet	2131	Middle Bronze Age		
2801	Prehistorian Indet	2136	Middle Bronze Age		
2831	Prehistorian Indet	2161	Middle Bronze Age		
2851	Prehistorian Indet	2181	Middle Bronze Age		
2861	Prehistorian Indet	2223	Middle Bronze Age		
2881	Prehistorian Indet	2261	Middle Bronze Age		
2913	Prehistorian Indet	2271	Middle Bronze Age		
2931	Prehistorian Indet	2331	Middle Bronze Age		
2951	Prehistorian Indet	2341	Middle Bronze Age		
2971	Prehistorian Indet	2361	Middle Bronze Age		
2981	Prehistorian Indet	2371	Middle Bronze Age		

3181	Prehistorian Indet	2381	Middle Bronze Age		
3201	Prehistorian Indet	2391	Middle Bronze Age		
		2411	Middle Bronze Age		
		2421	Middle Bronze Age		
		2466	Middle Bronze Age		
		2471	Middle Bronze Age		
		2521	Middle Bronze Age		
		2551	Middle Bronze Age		
		2553	Middle Bronze Age		
		2581	Middle Bronze Age		
		2591	Middle Bronze Age		
		2631	Middle Bronze Age		
		2641	Middle Bronze Age		
		2660	Middle Bronze Age		
		2662	Middle Bronze Age		
		2664	Middle Bronze Age		
		2681	Middle Bronze Age		
		2691	Middle Bronze Age		
		2692	Middle Bronze Age		
		2693	Middle Bronze Age		
		2711	Middle Bronze Age		
		2731	Middle Bronze Age		
		2761	Middle Bronze Age		
		2771	Middle Bronze Age		
		2792	Middle Bronze Age		
		2811	Middle Bronze Age		
		2821	Middle Bronze Age		
		2836	Middle Bronze Age		
		2871	Middle Bronze Age		
		2891	Middle Bronze Age		
		2911	Middle Bronze Age		

		2921	Middle Bronze Age		
		3001	Middle Bronze Age		
		3011	Middle Bronze Age		
		3021	Middle Bronze Age		
		3031	Middle Bronze Age		
		3036	Middle Bronze Age		
		3041	Middle Bronze Age		
		3051	Middle Bronze Age		
		3052	Middle Bronze Age		
		3061	Middle Bronze Age		
		3071	Middle Bronze Age		
		3081	Middle Bronze Age		
		3082	Middle Bronze Age		
		3091	Middle Bronze Age		
		3096	Middle Bronze Age		
		3098	Middle Bronze Age		
		3101	Middle Bronze Age		
		3111	Middle Bronze Age		
		3121	Middle Bronze Age		
		3131	Middle Bronze Age		
		3136	Middle Bronze Age		
		3141	Middle Bronze Age		
		3151	Middle Bronze Age		
		3161	Middle Bronze Age		
		3171	Middle Bronze Age		
		3191	Middle Bronze Age		
		3196	Middle Bronze Age		
		3198	Middle Bronze Age		
		3211	Middle Bronze Age		

2. Supplementary Table S2. Taxonomical Representation in NISP According to SU.

NISP	*Bos taurus*	*Equus ferus*	*Ovis aries*	*Capra hircus*	*Ovis Capra*	*Canis*	*Sus dom*	*Sus scrofa*	*Lepus*	*Orycto.*	*Cervus*	Large Indet size	Large-Intermed. Size	Intermed-Small indet Size	Small Size	partridge	crow	Barn owl	Bird Indet	Bufo bufo	Indet	Total
**1001**															6							6
**1011**	1				12							1			8						1	23
**1016**	3			1	1		1					1			10							17
**1018**					4	55						2										61
**1021**			2									1			3							6
**1031**	1				2							15										18
**1041**	2																					2
**1061**	2				5							1									3	11
**1071**					4		1					7			1							13
**1081**	11										1	3									8	23
**1091**	6					1	13			1		4			8							33
**1101**	3				1							9			4							17
**111**	3				1																	4
**1111**	11																				8	19

**1121**												7										7
**1161**												1										1
**1666**															4		45					49
**1171**					6																23	29
**1181**	1				4	42	4															51
**1191**												1									3	4
**1201**	3				1							10			3							17
**1202**					2																7	9
**1204**	1				2							5										8
**121**	3		1		5							2										11
**1221**	11				1							1			1							14
**1222**	2				4							5									10	21
**1231**	1																					1
**1241**	21				2							30			9							62
**1251**	1		2	1	48		12			23		2			57	4						150
**1271**		1																				1
**1281**	1																					1
**1301**												15										15
**1306**					1																	1

**1311**						1						6										7
**1321**	2											20										22
**1323**			1		1	2						3										7
**1331**	14		2		28		39			4		80			18						80	265
**1336**	71		4		88	2	7			2		176			221						2	573
**1341**	4			1	4	13						9										31
**1351**	33				7							5			6							51
**1361**	129			1	4				1			70			43			28				276
**1371**															5							5
**1381**	1														1							2
**1391**	1				3							2										6
**1401**					1										7							8
**1402**	1																					1
**141**					1										3							4
**1411**					5							2			5							12
**1423**	8				1		2					9										20
**1431**	24				1		3					12										40
**1436**	2				5							9			5							21
**1441**	8				10							7			6							31

**1451**					10										14							24
**1461**	28						120					4			20						6	178
**1470**	4				4	12						5										25
**1476**	2		1		4	1	5					2									3	18
**1501**					2		2								5							9
**1511**							1					5										6
**1521**													6									6
**1531**	1																					1
**1541**	3					1	1					8			10							23
**1551**	1				1							2										4
**1561**	3					1				1		11										16
**1581**	4				4										8							16
**1591**															1							1
**1601**	4	4		5	4							1			4							22
**1611**	1																					1
**1621**	2				2					1												5
**1641**	7			2	6							4										19
**1651**												4			5						4	13
**1661**	2					31	3					1			2						7	46

**1662**							232															232
**1663**						233																233
**1664**				174																		174
**1665**	4				3																2	9
**1681**															7							7
**1691**	9		3		2		1					1										16
**1701**															2							2
**1711**															7							7
**1731**	2				3																4	9
**1736**	1				1										4							6
**1751**					1																3	4
**1761**															1							1
**1771**					1							1										2
**1781**															3							3
**1791**	4											3										7
**1796**							2															2
**1811**	6																					6
**1821**								1				1			3							5
**1831**	24						4															28

**1836**												1										1
**1841**										1		5			4						10	20
**1851**	2				3							3										8
**1861**	3		1		2		4			1		5			5						6	27
**1881**	1																					1
**1891**					2					1		4										7
**1893**	4				1							3										8
**1895**	1											2			20							23
**1961**	1				1							1			12							15
**191**	4				4										4						1	13
**1921**					6										10							16
**1923**					1										3						3	7
**1931**				2											2							4
**1941**			8																			8
**1951**	1				2																1	4
**196**															4							4
**1976**	4				1								1		9							15
**1981**	10		1		6	3	5					5			5						1	36
**1991**												2										2

**2001**					6							1			3							10
**962**	1														2						6	9
**2051**					2							20			1							23
**201**												2										2
**2011**		1	1		1							1										4
**2262**					1										1							2
**2081**	3			1								14										18
**2091**					2																2	4
**211**															2							2
**2111**					1																	1
**2131**	1				4						1	6			6							18
**2136**	2				3							1			3							9
**2141**															1							1
**2156**	1																					1
**2161**	6						6					7										19
**2181**	1	4		1	6							2										14
**2201**	15	7		4	23		18			1		51			75						3	197
**2203**	10														7							17
**221**					1																1	2

**2221**			4																			4
**2223**	5			6		35																46
**2227**	1				1							2									4	8
**2236**	7	6	1		1	1	3				1	43		1								64
**2251**			1		2										5					18		26
**2261**	5		4		1																	10
**2262**	3				12							2										17
**2271**				6	9		2					3			1							21
**2281**	2																					2
**2301**	2			1	2							8			7							20
**2321**												4										4
**2331**	7				9		2					14			14							46
**2341**	3				2							3			3							11
**2361**					2							1			3							6
**2371**	5			1	10	3	23			4		17			20						42	125
**2381**	6				2		1					3										12
**2391**	55	4			13		48	8			1	74									10	213
**2396**	13			1	11		12			1		2	1		31							72
**2401**	1											1			2							4

**241**	2																				6	8
**2411**	10			1	7	4	1			3		3			20							49
**2414**					4																	4
**2417**	6	3	1	1	20							64			1						15	111
**2420**	4				2							8										14
**2441**	18				5							2										25
**2451**															2							2
**2461**	1		6		1					1		5			3							17
**2466**					2								1								3	6
**2491**	1				4										11							16
**251**															4							4
**2521**	2				12					2	1	1			23							41
**2540**															1							1
**2551**	6			3	12							2			5							28
**2553**	9				1	4	1															15
**2557**					1										4							5
**2561**	3		2		12							4									4	25
**2565**												1										1
**2581**	4				9	2						9			7							31

**2591**	9			7	2							8										26
**2601**	1		3		3										1							8
**2631**	5				3		1															9
**2641**	11		2	1	20	1			2	3		1			15						1	57
**2651**																					7	7
**2652**			1		3							12			7							23
**2653**			2		8										4							14
**2660**			1									4										5
**2662**	1			1	6						1	9			13							31
**2664**						5	1					1										7
**2666**	2											1										3
**2681**					19				2	3									1		8	33
**2691**	2				7					1		2			14							26
**2692**				182																		182
**2693**	1			33																		34
**2101**	4		1									10			2							17
**2711**						2						1			1							4
**2730**	7																					7
**2731**	1				11	1						3										16

**2743**															1							1
**2745**												7										7
**2351**	75									1		11										87
**2761**	4		2		1	1				1		2			3							14
**2776**					1		4								1							6
**2796**	5				3		3			1		5										17
**2799**		1																				1
**2811**	1				6					2		8										17
**2821**				1	1					1		13									7	23
**2823**		2		1	2			2		7		1			1							16
**2831**												1										1
**2841**	2	3					2					4									3	14
**2851**												1									4	5
**2861**		1																				1
**2871**	3		1	1	8					1		1			7							22
**2881**					2							1									2	5
**291**	1				1							1										3
**2911**			1		2							9			5							17
**2921**	10		3		4	1						12			1							31

**2931**							4								16							20
**2981**	2			5	6	6						6			2							27
**2961**															1							1
**2971**			2	1	18		2			1		5			16							45
**3001**	16				18		14			1		28			45							122
**3011**	1				3	29	9					5			5							52
**3021**	93		9	2	64		9			1		41			6						27	252
**3031**			1		3	2	1					1			1						5	14
**3036**	20				41					4		22			77						40	204
**3041**	1						2														4	7
**3051**	2						1					1			5							9
**3052**							4														3	7
**3061**	2				1	13	3					3										22
**3071**	2				3	1	1					2										9
**3081**	2				1							4			6							13
**3082**					3	3									2							8
**3091**	3				11							1			7							22
**3096**	6					12						10			5							33
**3098**	51					1	3					2									15	72

**310**	1														1							2
**3101**	19			1	28		11					3										62
**3111**						1						6			4							11
**3121**	14											12										26
**3131**	6											4			4							14
**3136**	6		7		7							2			1							23
**3141**	2				1	1	6					19									5	34
**3151**	1			1	1							4			3							10
**3161**					1																	1
**3171**	149		1		4		1															155
**3181**												5										5
**3191**					1	5						1			1							8
**3196**	1											1			3							5
**3198**					5																2	7
**3201**					1					1												2
**3211**	2											4			2							8
**351**	3						1														1	5
**356**	12						7					8									1	28
**361**			1	1								1			6							9

**371**					1							20										21
**381**	1																					1
**411**												3										3
**421**												10										10
**431**	8																					8
**451**					2							1			1							4
**461**	2			2	17	1						4										26
**471**				3	2																10	15
**481**	26											78										104
**491**															3							3
**511**	145				1										1						1	148
**521**	1																					1
**531**	2																					2
**541**					1										1							2
**856**	25				1						3	150			2							181
**571**	22	3	7	1	3		2					25			5							68
**573**	48											21									1	70
**801**	1				1																	2
**621**	1			1	1										2							5

**631**					7																	7
**636**					1		11			1												13
**651**												1										1
**661**	11		1																			12
**663**	1			31																		32
**671**															1							1
**681**			6									3										9
**701**												1										1
**702**					3							3										6
**721**	3																					3
**741**									1			8			5							14
**761**	6				1	3	1					4			10						32	57
**771**															2							2
**781**	6				6							5			4							21
**791**	2			1	2	1						12										18
**711**															4							4
**811**	1				2							1										4
**821**	5				6	1									8						2	22
**826**	3				6		1					5			24						1	40

**831**	12				1					1		6			6						3	29
**841**	3				8							9			7							27
**854**	1						1															2
**857**	6			192	2							6			1							207
**859**	12				18					4		15			47							96
**801**					2							7										9
**870**															1							1
**872**	9				11							52			10							82
**873**	1				1										1							3
**876**	4											19										23
**878**	10				1							11			9							31
**886**	2				3							7										
**901**												6										6
**911**					1										5							6
**931**							2								10							12
**941**			1	4	3																3	11
**961**					1										2							3
**963**	1																					1
**971**	3			7		1									15							26

**981**	83		2		2						1	2			2						40	132
**991**	3		2		10	3				1					14							33
**indet**												1									1	2
**Total**	1780	40	103	693	1020	542	686	11	6	83	10	1817	9	1	1374	4	45	28	1	18	521	8794

3. Supplementary Table S3. Taxonomical Representation in MNI According to SU.

MNI	*Bos taurus*	*Equus ferus*	*Ovis aries*	*Capra hircus*	*Ovi-Cap*	*Canis fam.*	*Sus dom*	*Sus scrofa*	*Lepus*	*Oryctol.*	*Cervus*	partridge	crow	Barn owl	Bufo bufo	Total
**1001**																-
**1011**	1				1											2
**1016**	1			1	1		1									4
**1018**					1	1										2
**1021**			1													1
**1031**	1				1											2
**1041**	1															1
**1061**	1				1											2
**1071**					1		1									2
**1081**	1										1					2
**1091**	1					1	1			1						4
**1101**	1				1											2
**111**	1				1											2
**1111**	1															1

**1121**																-
**1161**																-
**1666**													1			1
**1171**					1											1
**1181**	1				1	2	1									5
**1191**																-
**1201**	1				1											2
**1202**					1											1
**1204**	1				1											2
**121**	1		1		1											3
**1221**	1				1											2
**1222**	1				1											2
**1231**	1															1
**1241**	1				1											2
**1251**	1		1	1	1		2			2		1				9
**1271**		1														1
**1281**	1															1
**1301**																-

**1306**					1											1
**1311**						1										1
**1321**	1															1
**1323**			1		1	1										3
**1331**	1		1		1		2			1						6
**1336**	3		1		2	1	1			1						9
**1341**	1			1	2	2										6
**1351**	1				1											2
**1361**	3			2	1				1					1		8
**1371**																-
**1381**	1															1
**1391**	1				1											2
**1401**					1											1
**1402**	1															1
**141**					1											1
**1411**					1											1
**1423**	1				1		1									3
**1431**	1				1		1									3

**1436**	1				1											2
**1441**	1				1											2
**1451**					1											1
**1461**	1						3									4
**1470**	1				1	1										3
**1476**	1		1		1	1	1									5
**1501**					1		1									2
**1511**							1									1
**1521**																-
**1531**	1															1
**1541**	1					1	1									3
**1551**	1				1											2
**1561**	1					1				1						3
**1581**	1				1											2
**1591**																-
**1601**	1	1		1	1											4
**1611**	1															1
**1621**	2				2					1						5

**1641**	1			1	1											3
**1651**																-
**1661**	1					1	1									3
**1662**							1									1
**1663**						1										1
**1664**				1												1
**1665**	1				1											2
**1681**																-
**1691**	1		1		1		1									4
**1701**																-
**1711**																-
**1731**	1				1											2
**1736**	1				1											2
**1751**					1											1
**1761**																-
**1771**					1											1
**1781**																-
**1791**	1															1

**1796**							1									1
**1811**	1															1
**1821**								1								1
**1831**	1						1									2
**1836**																-
**1841**										1						1
**1851**	1				1											2
**1861**	1		1		1		1			1						5
**1881**	1															1
**1891**					1					1						2
**1893**	1				1											2
**1895**	1															1
**1961**	1				1											2
**191**	1				1											2
**1921**					1											1
**1923**					1											1
**1931**				1												1
**1941**			1													1

**1951**	1				1											1
**196**																-
**1976**	1				1											2
**1981**	1		1		1	1	1									5
**1991**																-
**2001**					1											1
**962**	1															1
**2051**					1											1
**201**																-
**2011**		1	1		1											3
**2262**					1											1
**2081**	1			1												2
**2091**					1											1
**211**																2
**2111**					1											1
**2131**	1				1						1					3
**2136**	2				1											2
**2141**																1

**2156**	1															1
**2161**	2						1									3
**2181**	1	1		1	1											4
**2201**	2	1		1	2		2			1						9
**2203**	1															1
**221**					1											1
**2221**			1													1
**2223**	1			1		2										4
**2227**	1				1											2
**2236**	2	1	1		1	1	1				1					8
**2251**			1		1										1	3
**2261**	1		1		1											3
**2262**	1				1											2
**2271**				1	1		1									3
**2281**	1															1
**2301**	1			1	1											3
**2321**																-
**2331**	1				1		1									3

**2341**	1				1											2
**2361**					1											1
**2371**	1			1	1	1	1			1						5
**2381**	1				1		1									3
**2391**	3	1			2		1	1			1					10
**2396**	1			1	1		1			1						5
**2401**	1															1
**241**	1															1
**2411**	1			1	1	1	1			1						6
**2414**					1											1
**2417**	1	1	1	1	1											5
**2420**	1				1											2
**2441**	2				1											3
**2451**																-
**2461**	1		1		1					1						4
**2466**					1											1
**2491**	1				1											2
**251**																-

**2521**	1				1					1	1					4
**2540**																1
**2551**	1			1	1											3
**2553**	1				1	1	1									4
**2557**					1											1
**2561**	1		1		1											3
**2565**																-
**2581**	1				1	1										3
**2591**	1			1	1											3
**2601**	1		1		1											3
**2631**	1				1		1									3
**2641**	1		1	1	1	1			1	1						7
**2651**																-
**2652**			1		1											2
**2653**			1		1											2
**2660**			1													1
**2662**	1			1	1						1					4
**2664**						1	1									2

**2666**	1															1
**2681**					1				1	1						4
**2691**	1				1					1						3
**2692**				1												1
**2693**	1			1												2
**2101**	1		1													2
**2711**						1										1
**2730**	1															1
**2731**	1				1	1										3
**2743**																-
**2745**																-
**2351**	1									1						1
**2761**	1		1		1	1				1						5
**2776**					1		1									2
**2796**	1				1		1			1						4
**2799**		1														1
**2811**	1				1					1						3
**2821**				1	1					1						3

**2823**		1		1	1			1		1						5
**2831**																-
**2841**	1	1					1									3
**2851**																-
**2861**		1														1
**2871**	1		1	1	1					1						5
**2881**					1											1
**291**	1				1											1
**2911**			1		1											2
**2921**	1		1		1	1										4
**2931**							1									1
**2981**	1			1	1	1										4
**2961**																-
**2971**			1	1	1		1			1						5
**3001**	2				1		1			1						5
**3011**	1				1	2	1									5
**3021**	2		1	1	2		1			1						8
**3031**			1		1	1	1									4

**3036**	1				1					1						3
**3041**	1						1									2
**3051**	1						1									2
**3052**							1									1
**3061**	1				1	1	1									4
**3071**	1				1	1	1									4
**3081**	1				1											2
**3082**					1	1										2
**3091**	1				1											2
**3096**	1					1										2
**3098**	1					1	3									3
**310**	1															1
**3101**	1			1	1		1									4
**3111**						1										1
**3121**	1															1
**3131**	1															1
**3136**	1		1		1											3
**3141**	1				1	1	1									4

**3151**	1			1	1											3
**3161**					1											1
**3171**	2		1		1		1									5
**3181**																-
**3191**					1	1										2
**3196**	1															1
**3198**					1											1
**3201**					1					1						2
**3211**	1															1
**351**	1						1									2
**356**	1						1									2
**361**			1	1												2
**371**					1											1
**381**	1															1
**411**																-
**421**																-
**431**	1															1
**451**					1											1

**461**	1			1	1	1										4
**471**				1	1											2
**481**	1															1
**491**																-
**511**	1				1											2
**521**	1															1
**531**	2															2
**541**					1											1
**856**	1				1						1					3
**571**	1	1	1	1	1		1									5
**573**	2															1
**801**	1				1											2
**621**	1			1	1											3
**631**					1											1
**636**					1		1			1						3
**651**																-
**661**	1		1													2
**663**	1			1												2

**671**																-
**681**			1													1
**701**																-
**702**					1											1
**721**	1															1
**741**									1							1
**761**	1				1	1	1									4
**771**																-
**781**	1				1											2
**791**	1			1	1	1										4
**711**																-
**811**	1				1											2
**821**	1				1	1										3
**826**	1				1		1									3
**831**	1				1					1						3
**841**	1				1											2
**854**	1						1									2
**857**	1			1	1											3

**859**	1				1					1						3
**801**					1											1
**870**				1												-
**872**	1				1											2
**873**	1				1											1
**876**	1															1
**878**	1				1											2
**886**	1				1											2
**901**																-
**911**					1											1
**931**							1									1
**941**			1	4	1											6
**961**					1											3
**963**	1															1
**971**	1			1		1										3
**981**	1		1		1						1					4
**991**	1		1		1	1				1						5
**Total**	193	13	41	47	178	47	67	3	4	35	8	1	1	1	1	637

**%**	30,1	2,0	6,4	7,4	27,9	7,4	10,4	0,5	0,6	5,5	1,3	0,2	0,2	0,2	0,2	

4 **Supplementary Tables S4–S6. Mortallity Pattern for *Bos, Ovis, Capra* and *Sus*.**

Supplementary Table S4. Mortallity Pattern for *Bos taurus*.

Bos taurus				
**Chronology**	**SU**	**Skeletal Unit**	**Age in months**	**Preliminary seasonal estimates**
Bell-Beaker Bronze Age	2201	Mandible and teeth	5-6	Autumn
	2417	Long Bones	48	Spring-Summer
	573	Mandible and teeth	22-24	Spring-Summer
Middle Bronze Age	3211	Radio	40	Summer-Autumm
	1366	Mandible and teeth	36	Spring-Summer
	3171	Mandible and Long Bone	16-18	Summer-Autumm
	3171	Long Bones	20	Autum-Winter
	3171	Long Bones	30	Autum
	3136	Mandible and teeth	15-18	Summer-Autumm
	3171	Radio	35?	Spring?
	2553	Mandible and teeth	5	Autum
	2631	Mandible and teeth	4-5	Autum
	2081	Tibiae	50	¿?

Supplementary Table S5. Mortallity Pattern for *Sus*.

Sus domesticus				
**Chronology**	**SU**	**Skeletal Unit**	**Age in months**	**Preliminary seasonal estimates**
Bell-Beaker Bronze Age	1251	Long Bone	42	Autum
	2201	Mandible	19-21	Autum-Winter
	2201	Maxillar	8-10	Winter
Middle Bronze Age	2371	Long Bone	o	Winter
	1461	Long Bone	0-2	Sppring
	1461	Mandible and Maxillar	14-17	Summer-Autumm
	1461	Mandible	4 es	Summer
	1461	Maxillar	18-19	Autum
	1461	Maxillar	19-21	Winter
	1431	Mandible	14-17	Summer-Autumm
	3011	Mandible	17-19	Autum
	3011	Mandible	10-11	Winter
	1476	Maxillar	6-8	Autum-Winter
	1423	Phalange	18?	Autum-Winter
	1091	Mandible	19-21	Winter
	1331	Mandible and Maxillar	19-21	Winter
	2331	Maxillar	17	Autum
	1662	All skeletal	19-21	Winter

Supplementary Table S6. Mortallity Pattern for *Ovis* and *Capra*.

Ovis aries				
**Chronology**	**SU**	**Skeletal Unit**	**Age in months**	**Preliminary seasonal estimates**
Bell-Beaker Bronze Age	1251	M1 Lower	5	Autum
	1251	M1 Lower	6	Autum
Middle Bronze Age	2461	Mandible	9	Winter
	571	P4 Lower	18	Autum
	2761	P4 Lower	6	Autum
**Chronology**	**SU**	*Capra hircus*		
Bell-Beaker Bronze Age	1251	M1 Lower	17	Autum
Middle Bronze Age	1664	All skeletal	1-3	Summer-Autumm
	2693	All skeletal	1-3	Summer-Autumm
	2641	M3 Lower	18	Autum
	471	M2 Lower	6-7	Autum
	2223	M1 Lower	23-24	Winter
	441	M1 Lower	23-24	Winter
	571	M2 Lower	4-5	Autum
	941	M1 Lower	26-30	Summer-Autumm?
	791	M1 Lower	25-30	Summer-Autumm?
**Chronology**	**SU**	***OVIS / CAPRA***		
Bell-Beaker Bronze Age	1251	M3 upper	18	Autum
	1251	P4 Lower	5	Autum
	2811	M3 upper	9	Winter
	991	Maxillar	7	Autum
	3021	M2 upper	6	Autum
	2414	M2 upper	6	Autum
	2776	All skeletal	1-3	Spring-Summer

5 Supplementary Tables S7 and S8. Carnivore tooth marks for the Bronze Age of Pista de Motos

Supplementary Table S7. Carnivore tooth marks –TM- for Pista de Motos.

NISP with tooth marks	*Equus*	*Bos*	*Canis*	*Ovis / Capra*	*Ovis*	*Capra*	*All Ovis and Capra*	*Sus*	*Cervus*	*Lepus*	*Oryct.*	Large Size	Small size	Indet
Horn		1				0	0							
Craneal		0	0	3		0	3	0			0	2	0	0
Mandible	0	0	0	3	0	1	4	0			0	1	0	
Vertabrae	0	2	0	3	0	1	4	1			0	1	0	0
Rib	0	12	2	4	0	1	5	2				0	0	
Scapule	1	5		2	0	0	2	1		0	0	0	0	
Humerus	1	10		5	1	0	6	4			0	0	2	
Radio	0	6	2	6	1	3	10	0	1	0	0		0	
Ulna	0	3	1	1		0	1	1			0			
Metacarpal		8		3	3		6		0					
Pelvis	0	3		1	0	0	1	0	0	0	1	0		
Femur	0	3		3	0	0	3	2	0		0	1	0	
Tibia	1	7	1	15	0		15	3	0	1	0	1	0	
Metatarsal	0	9		6	0	1	7		0				0	
Metapodial	0	2	0	1			1	0	0		1	0		
Talus		3			0	0	0	0						
Calcaneous	0	2	0	0	0	0	0		0		0			
Phalange	0	3		0			0	1						
indet.				0			0					33	41	2
Total	3	79	6	56	5	7	68	15	1	1	2	39	43	2

Supplementary Table S8. % Carnivore tooth marks –TM- for Pista de Motos.

% NISP with tooth marks	*Equus*	*Bos*	*Canis*	*Ovis /Capra*	*Ovis*	*Capra*	*All Ovis and Capra*	*Sus*	*Cervus*	*Lepus*	*Oryct.*	Large Size	Small size	Indet
Horn		1.1												
Craneal				3.8			3.5					1.7		
Mandible				6.4		5.0	5.6					6.7		
Vertabrae		0.7		4.4		0.4	1.6	1.5				2.9		
Rib		5.1	3.9	1.2		0.7	1.1	2.3						
Scapule	100.0	21.7		6.7			4.7	25.0						
Humerus	50.0	22.7		12.5	25.0		10.5	36.4					28.6	
Radio		9.4	25.0	24.0	12.5	12.0	17.2		100.0					
Ulna		10.3	12.5	8.3			4.5	7.7						
Metacarpal		25.0		37.5	37.5		27.3							
Pelvis		17.6		5.0			3.3				9.1			
Femur		13.0		13.6			10.0	16.7				50.0		
Tibia	25.0	13.5	12.5	26.3			23.1	33.3		50.0		10.0		
Metatarsal		37.5		17.1		11.1	14.9							
Metapodial		15.4		10.0			10.0				6.7			

Talus		15.0												
Calcaneous		14.3												
Phalange		3.7						1.4						
indet.												2.2	3.3	0.4
Total	8.3	5.3	1.7	6.5	10.6	0.8	4.5	3.3	10.0	16.7	2.4	2.2	3.1	0.4

6 Supplementary Table S9. Burn bones.

SU	*Bos*	*Capra*	*Oryctolagus*	*Equus*	Large Size animal	Indet	*Lepus*	*Ovis / Capra*	*Ovis*	Small Size Animals	*Sus dom.*	Burn Bones NISP	NISP Total
1101	3				9			1		1		14	17
1221										1		1	14
1222								1				1	21
1331	1				1							2	265
1336					4							4	573
1361	3				1		1					5	248
1401								1				1	8
1651					4	4				4		12	13
1681										4		4	7
1841										1		1	20
1961										4		4	15
1921										1		1	16
1981								1				1	36
962						5				1		6	9
2111								1				1	4
2161	1											1	19
2201				3				1				4	197
2227	1				3	4		1				9	9
2236	1											1	64
2301										3		3	20
2371			4					1				5	102
2391	2				2							4	213

2396	1											1	72
2411	1		3					1				5	49
2417					8							8	111
2491	1							4		1		6	16
2521	1							3		6		10	41
2561	2					4		9	2			17	25
2652					12			3	1	7		23	23
2351	8											8	87
2823		1										1	16
2911					9				1			10	17
2981	2				1							3	27
2961										1		1	1
3021	11				2	16						29	252
3081	2				1			1		3		7	13
3082										2		2	8
3091	1											1	22
3098						5						5	72
3171	21											21	155
351	3										1	4	8
461		1						3				4	26
573	7											7	70*
761	1											1	57
826								1				1	40
859								1				1	96
801								2				2	9
878					3							3	29
901					6							6	6
981										2		2	132
Total	74	2	7	3	66	38	1	36	4	42	1	274	

1. **Supplementary Tables S10 and S11. Bones with cut marks**

Suplementary Table S10 Bones with cut marks.

NISP with cut mark	*Equus ferus*	*Bos taurus*	*Canis familiaris*	*Ovis/Capra*	*Ovis aries*	*Capra hircus*	*All Ovis/Capra*	*Sus domesticus*	*Sus scrofa*	*Cervus elaphus*	*Lepus sp*	*Oryct. cunic.*	**Large Size Mammal**	**Small Size Mammal**	**Indet**
Horn		1				0	0								
Craneal		1	0	1		0	1	2				0	3	4	0
Maxillar		1	0	1			1	0	0				0		
Mandíble	0	8	2	2	0	1	3	2				0	3	0	
Vertabrae	0	9	0	1	0	1	2	0				0	1	2	0
Rib	0	26	2	27	0	1	28	2					8	4	
Scapule	0	6		5	2	0	7	1			0	0	1	0	
Indet Upper Limb	0	0		0			0						2		
Humerus	0	13		5	1	1	7	2				0	8	1	
Radio	0	18	0	5	0	3	8	1	0	0	0	0		0	
Ulna	1	5	0	2		1	3	1	0			0			
Metacarpal		4		0	0		0			0					
Carpal		1											0		
Pelvis	0	3		1	0	1	2	0		0	0	0	0		
Femur	0	3		3	1	0	4	4		1		0	1	0	
Tibia	0	8	0	7	0		7	3	0	1	1	2	1	0	
Metatarsal	0	3		2	1	0	3			0				0	
Talus		6			0	0	0	0	1						
Calcaneous	0	2	0	1	1	0	2			1		0			
Phalange	0	23		0			0	0							
indet.				9			9						37	29	3

Total	1	141	4	72	6	9	87	18	1	3	1	2	65	40	3

Suplementary Table S11 Frequencies of bones with cut marks.

% NISP with cut mark	*Equus ferus*	*Bos taurus*	*Canis familiaris*	*Ovis/Capra*	*Ovis aries*	*Capra hircus*	*All Ovis/Capra*	*Sus domesticus*	*Sus scrofa*	*Cervus elaphus*	*Lepus sp*	*Oryct. cunic.*	**Large Size Mammal**	**Small Size Mammal**	**Indet**
Horn		1.1													
Craneal		0.4		1.3			1.2	3.2					2.6	4.3	
Maxillar		3.8		10.0			5.0						0.0		
Mandíble		12.1	8.7	4.3		5.0	4.2	8.7					20.0		
Vertabrae		3.0		1.5		0.4	0.8						2.9	15.4	
Rib		11.1	3.9	8.2		0.7	6.3	2.3					14.0	30.8	
Scapule		26.1		16.7	66.7		16.3	25.0					20.0	0.0	
Indet Upper Limb													33.3		
Humerus		29.5		12.5	25.0	7.7	12.3	18.2					40.0	14.3	
Radio		28.1		20.0		12.0	13.8	9.1						0.0	
Ulna	50.0	17.2		16.7		10.0	13.6	7.7							
Metacarpal		12.5													
Carpal		1.9													
Pelvis		17.6		5.0		11.1	6.7								
Femur		13.0		13.6	100.0		13.3	33.3		50.0			50.0		
Tibia		15.4		12.3			10.8	33.3		100.0	50.0	13.3	10.0		
Metatarsal		12.5		5.7	33.3		6.4								
Talus		30.0							50.0						
Calcaneous		14.3		33.3	100.0		18.2			100.0					

Phalange		28.0													
indet.				18.4			18.4						2.4	2.4	0.6
Total	2.8	9.5	1.2	8.4	12.8	1.1	5.8	4.0	16.7	30.0	16.7	2.4	3.6	2.9	0.6

8. **Supplementary Tables S12–S18. Biometric Analysis**

Supplementary Table S12. Biometric estimation for *Bos Taurus.* Measurements in mm according nomenclature of Driesh (1976)

Ref: **Driesch - 1976 A Guide To The Measurement of Animal Bones From Archaeological Site**

Animal	Bone	GL	DP	BP	DD	BD
Bos taurus	Humerus	0	35	61	0	
Bos taurus	Humerus	0	0	0	60	59
Bos taurus	Humerus					71
Bos taurus	Radio	0	41	82	0	
Bos taurus	Radio	0	39	78	0	
Bos taurus	Radio	0	36	67		
Bos taurus	Radio	0	36	37	0	
Bos taurus	Radio	0	35	67	0	
Bos taurus	Radio	0	35	61	0	
Bos taurus	Radio	0	35	61	0	
Bos taurus	Radio	0	33	67	0	
Bos taurus	Radio	0	32	66		
Bos taurus	Radio	0	30	67	0	
Bos taurus	Radio	0	30	65	0	
Bos taurus	Radio	0	30	62	0	
Bos taurus	Radio	0	27	49	0	
Bos taurus	Radio	0	22	68	0	
Bos taurus	Radio	0	40	0	0	
Bos taurus	Radio	0	36		0	
Bos taurus	Radio	0	0	60	0	
Bos taurus	Radio	0	0	0	37	62
Bos taurus	Radio	0	0	0	36	52
Bos taurus	Radio	0	0	0	34	58
Bos taurus	Femur	262				
Bos taurus	Tibia	0	0	0	37	64
Bos taurus	Tibia	0	0	0	36	52
Bos taurus	Tibia	0	0	0	36	58
Bos taurus	Tibia	367	0	0	48	65
Bos taurus	Tibia	330	75	81	40	60
Bos taurus	Tibia	235			76	48
Bos taurus	Tibia	235			76	48
Bos taurus	Tibia	0	0	0	46	
Bos taurus	Tibia	0	0	0	43	
Bos taurus	Tibia	0	0	0	41	
Bos taurus	Tibia	0	0	0	41	
Bos taurus	Tibia	0	0	0	40	
Bos taurus	Tibia	0	0	0	40	
Bos taurus	Tibia	0	0	0	36	
Bos taurus	Tibia	0	0	0	33	
Bos taurus	Tibia	0	0	0	33	
Bos taurus	Tibia	0	0	0	25	

Bos taurus	Tibia	0	35	61		
Bos taurus	Tibia	0	32	44		
Bos taurus	Patella	60				
Bos taurus	Patella	46				
Bos taurus	Talus	63				
Bos taurus	Talus	62				
Bos taurus	Talus	62				
Bos taurus	Talus	61				
Bos taurus	Talus	60				
Bos taurus	Talus	60				
Bos taurus	Talus	60				
Bos taurus	Talus	60				
Bos taurus	Talus	60				
Bos taurus	Talus	57				
Bos taurus	Talus	57				
Bos taurus	Talus	55				
Bos taurus	Talus	53				
Bos taurus	Calcaneous	135				
Bos taurus	Metacarpal	207	33	55	31	58
Bos taurus	Metacarpal	0	35	54	0	
Bos taurus	Metacarpal	0	34	55	31	
Bos taurus	Metacarpal	0	30	51	0	
Bos taurus	Metacarpal	0	30	51	0	
Bos taurus	Metacarpal	0	30	46	0	
Bos taurus	Metacarpal	0	27	50		
Bos taurus	Metacarpal	0	25	42	0	
Bos taurus	Metacarpal	0	23	47		
Bos taurus	Metacarpal	0	21	45	0	
Bos taurus	Metacarpal	0	0	0	36	
Bos taurus	Metacarpal	0	0	0	29	
Bos taurus	Metacarpal	0	0	0	29	
Bos taurus	Metacarpal	0	0	0	23	
Bos taurus	Metatarsal	214	40	41	25	50
Bos taurus	Metatarsal	0	49	50	0	
Bos taurus	Metatarsal	0	44	41		
Bos taurus	Metatarsal	0	0	0	40	
Bos taurus	Metatarsal	0	0	0	40	
Bos taurus	Metatarsal	0	0	0	24	
Bos taurus	Phalange 1	65	35	28	0	0
Bos taurus	Phalange 1	61			0	0
Bos taurus	Phalange 1	60	38	31	24	32
Bos taurus	Phalange 1	60	35	30	20	29
Bos taurus	Phalange 1	60	31	27	16	25
Bos taurus	Phalange 1	59	34	32	0	0
Bos taurus	Phalange 1	59	31	27	0	0
Bos taurus	Phalange 1	58	30	27	20	25
Bos taurus	Phalange 1	58				
Bos taurus	Phalange 1	58				

Bos taurus	Phalange 1	56	33	23	20	26
Bos taurus	Phalange 1	55	35	30	19	30
Bos taurus	Phalange 1	55	30	27	17	27
Bos taurus	Phalange 1	55	30	18	26	23
Bos taurus	Phalange 1	55	28	25	15	23
Bos taurus	Phalange 1	54	0	0	0	0
Bos taurus	Phalange 1	52	30	26	20	33
Bos taurus	Phalange 1	52	26	24	0	22
Bos taurus	Phalange 1	52	0	0	0	0
Bos taurus	Phalange 1	50	29	24	16	20
Bos taurus	Phalange 1	50	26	24	16	21
Bos taurus	Phalange 1	50	25	25	17	24
Bos taurus	Phalange 1	50	25	23	25	20
Bos taurus	Phalange 1	50	25	17	22	22
Bos taurus	Phalange 2	41	33	30	30	27
Bos taurus	Phalange 2	40	30	27	30	24
Bos taurus	Phalange 2	37	30	26	26	22
Bos taurus	Phalange 2	37	27	27	27	23
Bos taurus	Phalange 2	36	31	28	30	23
Bos taurus	Phalange 2	36	25	24	30	24
Bos taurus	Phalange 2	35	30	29	30	24
Bos taurus	Phalange 2	35	30	21	25	22
Bos taurus	Phalange 2	35	29	28	23	23
Bos taurus	Phalange 2	33	27	25	26	22
Bos taurus	Phalange 2	32	28	25	24	22
Bos taurus	Phalange 2	31	24	20	20	18
Bos taurus	Phalange 2	30	26	21	22	18
Bos taurus	Phalange 2	30	25	23	15	19
Bos taurus	Phalange 2	30	24	22	20	19
Bos taurus	Phalange 2	30	0	0	0	0
Bos taurus	Phalange 3	65				
Bos taurus	Phalange 3	64				
Bos taurus	Phalange 3	63				
Bos taurus	Phalange 3	62				
Bos taurus	Phalange 3	60				
Bos taurus	Phalange 3	59				
Bos taurus	Phalange 3	57				

Supplementary Table S13. Estimated height at withers based on complete long bones

Bos taurus		
Bone	GL	**Estimated heigh (cm)**
Femur	262	116.9
Tibia	367	126.6
Tibia	330	113.8
Metacarpal	207	129.3 (Ox)
Metatarsal	214	118.7

Supplementary Table S14. Biometric estimation for or *Sus domesticus and Sus scrofa*.. Measurements in mm according nomenclature of Driesh (1976).

Animal	Bone	**GL**	**DP**	**BP**	**DD**	**BD**	**Estimated height (cm)**
Sus scrofa	Talus	50	0	0	0	0	+75
Sus scrofa	Talus	40	0	0	0	0	74
Sus scrofa	Radio	0	21	30	0	0	
Sus scrofa	Tibia	0	0	0	24	29	
Sus domesticus	Tibia	167	30	35	20	27	
Sus domesticus	Radio	120	15	22	20	25	-70 cm
Sus domesticus	Radio	115			0	0	< 73 cm
Sus domesticus	Calcaneous	62					62 cm
Sus domesticus	Talus	40	0	0	0	0	74 cm
Sus domesticus	Patella	30					
Sus domesticus	Radio	0	17	24	0	0	
Sus domesticus	Humerus	0	0	0	35	29	
Sus domesticus	Tibia	0	0	0	17	22	

Supplementary Table S15. Biometric estimation for *Equus ferus.* Measurements in mm according nomenclature of Driesh (1976).

Animal	Bone		GL	DP	BP	DD	BD
Equus ferus	Phalange 1		65	0	0	0	0
Equus ferus	Phalange 1		50	0	0	0	0
Equus ferus	Phalange 2		42	35	23	45	41
Equus ferus	Femur		0	27	50		
Equus ferus	Tibia		0	0	0	45	65
Equus ferus	Tibia		0	0	0	44	62
Equus ferus	Radio		0	0	0	33	55

Supplementary Table S16. Biometric estimation for *Canis familiaris.* Measurements in mm according nomenclature of Driesh (1976).

Bone	GL	DP	BP	DD	BD	**Estimated height (cm)**
Tibia	0	0	0	15	20	
Tibia	0	0	0	15	20	
Tibia	159	24	20	12	15	46.4 cm
Fémur	150			23	20	45.1cm

Húmero	134	26	18	20	20	45.1 cm

Supplementary Table S17. Biometric estimation for *Capra pyrenaica and Capra hircus.* Measurements in mm according nomenclature of Driesh (1976).

Animal	Bone	**GL**	**DP**	**BP**	**DD**	**BD**	**Estimated height (cm)**
Capra pyrenaica	Radio	150	28	44			59.7
Capra pyrenaica	Metacarpal	140	21	35			72.1
Capra hircus	Tibia	204	40	35	17	21	60.5
Capra hircus	Femur	174	20	37	40	35	59.2
Capra hircus	Humerus	161	40	36	24	28	62.1
Capra hircus	Radio	150	11	25	16	26	59.7
Capra hircus	Metatarsal	116	14	17	13	21	61.9
Capra hircus	Metacarpal	105	16	20	15	24	60.3
Capra hircus	Calcaneous	47	0	0	0	0	
Capra hircus	Talus	25					
Capra hircus	Humerus	0	20	25	0	0	
Capra hircus	Metatarsal	0	19	19	0	0	
Capra hircus	Radio	0	15	23	0	0	
Capra hircus	Radio	0	14	26	0	0	
Capra hircus	Radio	0	14	26	0	0	
Capra hircus	Radio	0	12	25	0	0	
Capra hircus	Radio	0	12	24	0	0	
Capra hircus	Humerus	0	0	0	21	25	
Capra hircus	Humerus	0	0	0	21	25	
Capra hircus	Radio	0	0	0	16	23	
Capra hircus	Metatarsal	0	0	0	15	23	

Supplementary Table S18. Biometric estimation for *Ovis aries.* Measurements in mm according nomenclature of Driesh (1976).

Animal	Bone	**GL**	**DP**	**BP**	**DD**	**BD**
Ovis aries	Talus	30	0	0	0	0
Ovis aries	Tibia	0	0	0	16	22
Ovis aries	Metacarpal	0	0	0	0	21
Ovis / Capra	Tibia	0	17	24		

Ovis / Capra	Tibia	0	0	0	18	24
Ovis / Capra	Tibia	0	0	0	18	22
Ovis / Capra	Tibia	0	0	0	18	21
Ovis / Capra	Humerus	0	0	0	16	23
Ovis / Capra	Metacarpal	0	0	0	14	21
Ovis / Capra	Metatarsal	0	0	0	12	22
Ovis / Capra	Metatarsal	0	0	0	12	22

Supplementary Data S1. Bayesian Age Model.

```
Plot()
{
 Curve("IntCal20","intcal20.14c");
 Outlier_Model("General",T(5),U(0,4),"t");
 Sequence()
 {
  Boundary("Start Bell Beaker");
  Phase("Bell Beaker")
  {
   R_Date("SU1251",3768,31)
   {
    Outlier("General",0.05);
   };
   R_Date("SU2417",3764,25)
   {
    Outlier("General",0.05);
```

```
  };
 };
 Boundary("End Bell Beaker");
 Boundary("Start Middle Bronze Age");
 Phase("Middle Bronze Age")
 {
  R_Date("SU962",3269,21)
  {
   Outlier("General",0.05);
  };
  R_Date("SU2581",3161,27)
  {
   Outlier("General",0.05);
  };
  R_Date("SU1660",3090,24)
  {
   Outlier("General",0.05);
  };
 };
 Boundary("End Middle Bronze Age");
};
};
```
